# External validation and comparison of two variants of the Elixhauser comorbidity measures for all-cause mortality

**DOI:** 10.1371/journal.pone.0174379

**Published:** 2017-03-28

**Authors:** Yannick Fortin, James A. G. Crispo, Deborah Cohen, Douglas S. McNair, Donald R. Mattison, Daniel Krewski

**Affiliations:** 1 McLaughlin Centre for Population Health Risk Assessment, University of Ottawa, Ottawa, Ontario, Canada; 2 School of Epidemiology, Public Health and Preventive Medicine, University of Ottawa, Ottawa, Ontario, Canada; 3 Fulbright Canada Student, University of Pennsylvania, Philadelphia, Pennsylvania, United States of America; 4 Canadian Population Health Initiative (CPHI), Canadian Institute for Health Information (CIHI), Ottawa, Ontario, Canada; 5 Institute for Health Policy, Management and Evaluation, University of Toronto, Toronto, Ontario, Canada; 6 Cerner Corporation, Kansas City, Missouri, United States of America; 7 Risk Sciences International, Ottawa, Ontario, Canada; Monash University, AUSTRALIA

## Abstract

Assessing prevalent comorbidities is a common approach in health research for identifying clinical differences between individuals. The objective of this study was to validate and compare the predictive performance of two variants of the Elixhauser comorbidity measures (ECM) for inhospital mortality at index and at 1-year in the Cerner Health Facts® (HF) U.S. database. We estimated the prevalence of select comorbidities for individuals 18 to 89 years of age who received care at Cerner contributing health facilities between 2002 and 2011 using the AHRQ (version 3.7) and the Quan Enhanced ICD-9-CM ECMs. External validation of the ECMs was assessed with measures of discrimination [c-statistics], calibration [Hosmer–Lemeshow goodness-of-fit test, Brier Score, calibration curves], added predictive ability [Net Reclassification Improvement], and overall model performance [R^2^]. Of 3,273,298 patients with a mean age of 43.9 years and a female composition of 53.8%, 1.0% died during their index encounter and 1.5% were deceased at 1-year. Calibration measures were equivalent between the two ECMs. Calibration performance was acceptable when predicting inhospital mortality at index, although recalibration is recommended for predicting inhospital mortality at 1 year. Discrimination was marginally better with the Quan ECM compared the AHRQ ECM when predicting inhospital mortality at index (c_Quan_ = 0.887, 95% CI: 0.885–0.889 vs. c_AHRQ_ = 0.880, 95% CI: 0.878–0.882; p < .0001) and at 1-year (c_Quan_ = 0.884, 95% CI: 0.883–0.886 vs. c_AHRQ_ = 0.880, 95% CI: 0.878–0.881, p < .0001). Both the Quan and the AHRQ ECMs demonstrated excellent discrimination for inhospital mortality of all-causes in Cerner Health Facts®, a HIPAA compliant observational research and privacy-protected data warehouse. While differences in discrimination performance between the ECMs were statistically significant, they are not likely clinically meaningful.

## Introduction

With data on over 47 million unique patients who received care at nearly 500 US care facilities since 2000, the Cerner Health Facts® (HF) electronic health record database is a rich source of data available for epidemiological and health services research [1]. In addition to demographic and payer data, HF contains longitudinal diagnostic, procedure, pharmacy, and laboratory information on individuals receiving care within Cerner data networks.

To date, the predictive performance of commonly used comorbidity risk adjustment methods have yet to be corroborated in HF. Measures of comorbidity are useful tools for controlling for variation in overall patient health or adjusting for case-mix in epidemiological studies using electronic health data [2–5]. They are also used in observational drug effectiveness, health services, and outcomes studies when the unit of analysis cannot be appropriately randomized [6–10]. Measures of patient comorbidity have shown to be good predictors of short- and long-term mortality, hospital costs, length of stay (LOS), and readmission [11–14]. Failure to take patient comorbidity into account may lead to biased analyses, possibly due to confounding or systematic differences in health status among populations. Validated comorbidity measures may be used to address this issue.

In its simplest form, measures of comorbidity are aggregates of diagnostic codes used to identify the prevalence of predetermined health conditions in individuals documented in health data sources such as electronic health records. Of the many comorbidity measures validated for use with electronic health data, the original Elixhauser [15] comorbidity measures (ECM) are frequently reported as having greater predictive performance for short- and long-term mortality than competing models [11, 12, 16, 17]. The ECM target 30 medical, psychiatric, and lifestyle-related health conditions that are negatively associated with adverse health outcomes: congestive heart failure, cardiac arrhythmia, valvular disease, pulmonary circulation disorders, peripheral vascular disorders, hypertension (un/complicated), paralysis, neurological disorders, chronic pulmonary disease, uncomplicated diabetes, complicated diabetes, hypothyroidism, renal failure, liver disease, peptic ulcer disease without bleeding, aids/HIV, lymphoma, metastatic cancer, solid tumor without metastasis, rheumatoid arthritis/collagen vascular diseases, coagulopathy, obesity, weight loss, fluid and electrolyte disorders, blood loss anemia, deficiency anemia, alcohol abuse, drug abuse, psychoses, and depression. A few years after the publication of the original ECM, Quan et al. [18] and the Agency for Healthcare Research and Quality (AHRQ) [19] separately developed revised ECM variants, with Quan and colleagues reporting enhanced predictive performance for inhospital mortality compared to both the original ECM and the AHRQ ECM (version 3.0). Studies have not examined whether the Quan variant is superior to the most recent AHRQ ECM (version 3.7) at predicting inhospital mortality and inhospital mortality at 1-year despite differences in the ICD-9-CM codes used to identify prevalent health conditions by each variant. The AHRQ ECM also differs from the Quan ECM by the exclusion of diagnostic codes making up a cardiac arrhythmia health condition group. Neither the Quan nor the AHRQ ECMs have been validated in HF.

The objectives of this study were to conduct an external validation and compare the performance of the Quan and AHRQ (version 3.7) ECMs for predicting inhospital mortality of all-causes at index and at 1-year in HF.

## Methods

### Data source

Study data were derived from inpatient and emergency care encounters in Cerner Health Facts® (Kansas City, MO, USA), an administrative health database compliant with the US Health Insurance Portability and Accountability Act (HIPAA). Approximately 500 health care facilities have contributed patient-level clinical encounter data to HF since January 2000. Data contributors range in size from those with fewer than five beds to those with over 500 and are located throughout the U.S. with a greater proportion situated in the Northeastern region of the United States [20]. University affiliated teaching hospitals comprise 40% of data contributors and they contribute more than 60% of all health encounters. Contributing health care facilities are categorized by teaching status, population density, bed size, and census region. HF data includes diagnoses recorded during emergency department (ED) visits, outpatient care, and hospitalizations, pharmacy orders, surgical procedures, laboratory and microbiology tests, and clinical procedures [1].

This study was approved by the Office for Research Ethics and Integrity at the University of Ottawa.

### Study population and index encounter

Individuals 18 to 89 years of age at the time of an ED or inpatient encounter at any HF contributing facility between January 2002 and December 2012 were eligible for inclusion. One ED or inpatient encounter was randomly selected between January 1^st^, 2002 and December 31^st^, 2011 as the index encounter for each individual. Outpatient visits were excluded as possible index encounters since deaths, the study outcome, are relatively infrequent during outpatient care [21, 22]. Persons younger than 18 years were excluded due to the relatively lower prevalence of Elixhauser health conditions and mortality in this population. Individuals 90 years or older are assigned to a single category in HF in order to comply with HIPAA requirements and were excluded to ensure age remained continuous in our analyses. Care recipients who transferred to or from any other health facility during the index encounter were excluded to avoid cases with higher potential for missing information [23]. Patient characteristics included sex, age at the index encounter, health insurance status, and race restricted to Caucasians, African Americans, Hispanics, and Asians. Health insurance status was categorized using AHRQ recommendations [24], including private, Medicaid, Medicare, uninsured [self-pay], other (TRICARE-CHAMPUS, international plan, research funded, Title V, worker's compensation), or missing. For reasons unknown, some HF contributing health care facilities voluntarily withhold information on the health insurance status of their patients, which leads to a significant proportion (>40%) of missing values.

### Elixhauser comorbidity measures and variants

The original ECM [15] comprises binary indicators for the diagnosis of 30 clinical conditions, each defined by a combination of codes according to the International Classification of Disease, Ninth Edition (ICD-9) [25]. Two variants of the original ECM were compared with the study sample; the AHRQ’s latest comorbidity software (version 3.7) [19] and Quan et al.’s [18] Enhanced ICD-9-CM classification of comorbidities. The presence or absence of ECM comorbidities was assessed by examining the ICD-9-CM diagnostic codes recorded during the index encounter.

### Outcomes

The primary study outcomes were inhospital mortality of any causes during the index encounter and at 1-year. Inhospital mortality at 1-year was defined as a death recorded in a discharge abstract for an ED visit or an inpatient admission during the year that followed the admission date of the index encounter. Deaths recorded during the index encounter were therefore included in the mortality at 1-year outcome.

### Data analysis

The prevalence of comorbidities was described with counts and percentages while frequency differences between the ECMs were compared with McNemar’s test [26]. To quantify the size and the clinical importance of the observed differences in the prevalence of comorbidities across ECMs, we derived Cohen’s h [27]. Multiple logistic regression was used to predict the risk of mortality outcomes and output overall measures of model performance. External validation of the ECMs in HF was accomplished by deriving measures of calibration and discrimination for every predictive model, outcome, and sample combination considered [28, 29]. Model discrimination was assessed using the Area under the Receiver Operating Characteristic (ROC) curve (AUROC), an indicator of the ability of the ECMs to discriminate between the mortality statuses [30, 31]. The AUROC is often referred to as the concordance index number (c-statistic) and ranges between 0.5 [no discrimination] and 1.0 [perfect discrimination], with values above 0.7, 0.8, and 0.9 considered reasonable, strong, and exceptional, respectively [32]. The discrimination performance of each ECM was compared to 1) a baseline model, and 2) the competing ECM. Predictors in the baseline model were limited to sex and age at the index encounter to align with prior ECM validation and comparison studies [33, 34]. Differences in the AUROC between the fitted models were tested using the ROC and ROCCONTRAST statements in SAS [30]. The ROCCONTRAST option is an algorithm based on the non-parametric Mann-Whitney statistics developed by DeLong et al. [35] for comparing the significance of differences between correlated ROC curves.

Model calibration measures included the Hosmer–Lemeshow goodness-of-fit test, which evaluates the degree of agreement between the predicted and observed risk of inhospital mortality [36]. The Hosmer–Lemeshow goodness-of-fit test outputs a Pearson chi-square score with a corresponding p-value: rejection of the null hypothesis indicates an imperfect correlation between predicted and observed values. Calibration plots displaying predicted inhospital mortality probabilities on the x-axis and observed inhospital mortality frequencies on the y-axis were generated to visually inspect calibration performance across risk deciles. The plots were enhanced using a smoothing spline function. Brier scores, which equate to the mean squared difference between predicted probabilities and observed outcomes, were included as measures of model accuracy [with lower Brier scores reflecting greater accuracy] [37]. Explained variation was reported in terms of the adjusted R^2^.

It has been argued that the AUROC can be of limited value when comparing small incremental differences between predictive models [38]. To quantify the net improvement in predictive ability of the ECM that achieved the highest level of discrimination over the ECM with the lowest level of discrimination, we computed the net reclassification improvement (NRI) measure [39, 40]. Category-free NRI (NRI>0) assesses whether individuals are reclassified correctly in a prognosis model compared to a reference model. NRI>0 is a quantification of the net correct changes in model-based probabilities for both events [where improvement equates to increased probabilities of the outcome] and non-events [where improvement equates to decreased probabilities of the outcome] [41]. The NRI was implemented without risk categories because this approach allows for universal comparisons, it is robust against changing event-rated, and it was the most objective approach available in light of the insufficient evidence for meaningful risk categories for all-cause mortality in the literature [40]. NRI>0 values were computed using a SAS macro developed by Kennedy et al. [42] and are reported with the percentage of events and non-events correctly reclassified. Statistical analyses were completed with SAS 9.4 (SAS Institute Inc., Cary, NC, USA).

### Risk groups

Recent history of hospitalization or emergency department use is associated with increased risk of hospital readmission and death [43, 44]. To further explore the utility of the ECMs, measures of discrimination and calibration performance were generated for both high and low risk patient groups. Individuals with evidence of one or more inpatient stay in the 12 months preceding the index encounter, or three or more emergency department visits in the 3 months preceding the index encounter, were defined as high risk. Patients that did not satisfy the high-risk criteria were assigned to the low risk group.

### Sensitivity analyses

Admissible index encounters in this study included ED visits and inpatient stays. It is reasonable to hypothesize that persons admitted for inpatient stays would generally be at greater risk of inhospital death than persons visiting the emergency department. To investigate potential differences in ECM performance by index encounter type, complimentary validation analyses were performed on index encounters recorded as ED visits and inpatient stays separately.

## Results

We identified 3,273,298 unique individuals who satisfied our inclusion criteria and received care at a HF care facility between 2002 and 2011. Mean age was 43.9 years and women were the majority (53.8%) (Table 1). Individuals were primarily Caucasians (72.3%), with others identified as African American (21.7%), Hispanic (4.5%), or Asians (1.5%). Index encounters were reported by health care institutions from the four US census regions: Northeast (36.1%), Midwest (19.8%), South (32.9%), and West (11.2%). Privately insured individuals comprised 48.3% of non-missing payer class cases. Most patients in the sample were classified as low risk (92.0%). Approximately two-thirds of index encounters were ED visits (67.4%). A total of 31,298 (1.0%) and 50,215 (1.5%) inhospital deaths of all-cause were recorded during the index encounter and at 1-year, respectively. As expected, high risk patients had a greater frequency of inhospital mortality than low risk patients at index [1.9% vs 0.9%, χ^2^ (1, N = 3,273,298) = 2,792.1, p < .0001], and at 1 year [4.2% vs 1.3%, χ^2^ (1, N = 3,273,298) = 13,509.1, p < .0001].

**Table 1:** Patient demographic and index encounter characteristics, N = 3,273,298.

Characteristic	N (%)
**Sex**	
Female	1,761,525 (53.8)
Male	1,511,773 (46.2)
**Age (Years)**	
Mean ± SE	43.9 ± 0.01
**Race**	
Caucasian	2,366,665 (72.3)
African American	711,051 (21.7)
Hispanic	146,877 (4.5)
Asian	48,705 (1.5)
**Insurance Status**	
Private	795,449 (24.3)
Medicare	370,701 (11.3)
Medicaid	248,009 (7.6)
Uninsured	378,536 (11.6)
Other	139,745 (4.3)
Missing	1,340,858 (41.0)
**Risk Group**	
Low	3,010,916 (92.0)
High	262,382 (8.0)
**Inhospital Mortality**	
Deaths	31,298 (1.0)
**Inhospital Mortality at 1-Year**	
Deaths	50,215 (1.5)
**Care Setting, Index Encounter**	
ED Visit	2,204,680 (67.4)
Inpatient Stay	1,068,618 (32.6)
**Census Region**	
Northeast	1,180,270 (36.1)
Midwest	648,644 (19.8)
South	1,077,965 (32.9)
West	366,419 (11.2)

**Abbreviations:** ED, emergency department; SE, Standard error.

In descending order, hypertension, uncomplicated diabetes, chronic pulmonary disease, and fluid and electrolyte disorders were the most prevalent conditions identified by both ECMs (Table 2). Excluding HIV/AIDS, differences in prevalence between the ECMs for every health condition group were statistically significant based on McNemar’s test, p<0.0001. However, for most of the conditions assessed, the variation in prevalence between the ECMs differed by less than 1%, with the greatest differences observed for deficiency anemia (2.58%) and psychoses (1.22%). According to the interpretation criteria suggested by Cohen [27], only the deficiency anemia group demonstrated a small (h = 0.203) practically meaningful difference in prevalence by ECM.

**Table 2:** Prevalence of comorbid conditions by ECM variant, N = 3,273,298.

Condition	Quan, N (%)	AHRQ, N (%)	McNamar’s Test *P Value*	Cohen’s h
Hypertension	572,139 (17.48)	573,457 (17.52)	< .0001	0.001
Chronic Pulmonary Disease	256,170 (7.83)	248,676 (7.60)	< .0001	0.009
Diabetes Uncomplicated	226,807 (6.93)	227,815 (6.96)	< .0001	0.001
Fluid and Electrolyte Disorders	187,321 (5.72)	186,282 (5.69)	< .0001	0.001
Cardiac Arrhythmia	174,656 (5.34)	na	na	na
Depression	113,659 (3.47)	89,410 (2.73)	< .0001	0.043
Congestive Heart Failure	99,280 (3.03)	90,775 (2.77)	< .0001	0.015
Alcohol Abuse	89,460 (2.73)	86,307 (2.64)	< .0001	0.006
Hypothyroidism	85,493 (2.61)	83,926 (2.56)	< .0001	0.003
Obesity	79,680 (2.43)	80,996 (2.47)	< .0001	0.003
Other Neurological Disorders	76,237 (2.33)	97,360 (2.97)	< .0001	0.040
Drug Abuse	61,260 (1.87)	59,029 (1.80)	< .0001	0.005
Renal Failure	60,238 (1.84)	57,231 (1.75)	< .0001	0.007
Solid Tumor without Metastasis	56,376 (1.72)	56,519 (1.73)	< .0001	0.000
Valvular Disease	51,250 (1.57)	21,907 (0.67)	< .0001	0.087
Peripheral Vascular Disorders	41,651 (1.27)	43,410 (1.33)	< .0001	0.005
Diabetes Complicated	34,016 (1.04)	34,111 (1.04)	< .0001	0.000
Liver Disease	31,307 (0.96)	21,910 (0.67)	< .0001	0.032
Psychoses	29,656 (0.91)	69,664 (2.13)	< .0001	0.102
Coagulopathy	27,019 (0.83)	27,330 (0.83)	< .0001	0.001
Rheumatoid Arthritis/collagen	24,143 (0.74)	22,407 (0.68)	< .0001	0.006
Metastatic Cancer	22,836 (0.70)	23,004 (0.70)	< .0001	0.001
Weight Loss	21,219 (0.65)	19,429 (0.59)	< .0001	0.007
Pulmonary Circulation Disorders	21,084 (0.64)	20,894 (0.64)	< .0001	0.001
Deficiency Anemia	19,745 (0.60)	104,246 (3.18)	< .0001	0.203*
Paralysis	13,707 (0.42)	19,001 (0.58)	< .0001	0.023
Blood Loss Anemia	10,884 (0.33)	24,487 (0.75)	< .0001	0.058
Peptic Ulcer Disease excl. bleeding	8,251 (0.25)	507 (0.02)	< .0001	0.076
Lymphoma	6,778 (0.21)	6,920 (0.21)	< .0001	0.001
AIDS/HIV	4,246 (0.13)	4,246 (0.13)	1.000	0.000

* Described as a small effect size according to the interpretation criteria suggested by Cohen (1988).

**Fig 1:**
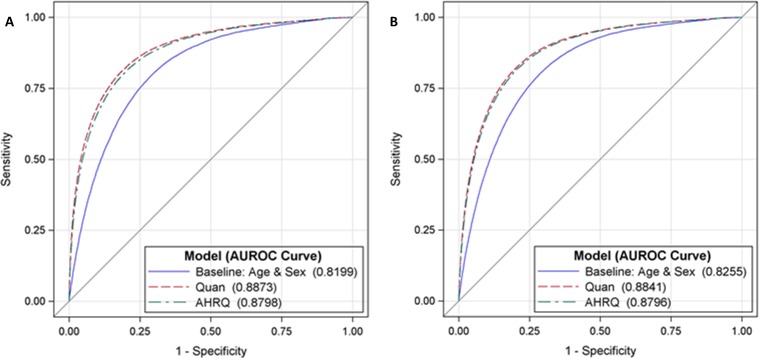
**AUROC comparisons by ECM for predicting inhospital mortality at index [A] and at 1 year [B].** ROC = receiver operating characteristic, AUROC = area under the receiver operating characteristic.

**Fig 2:**
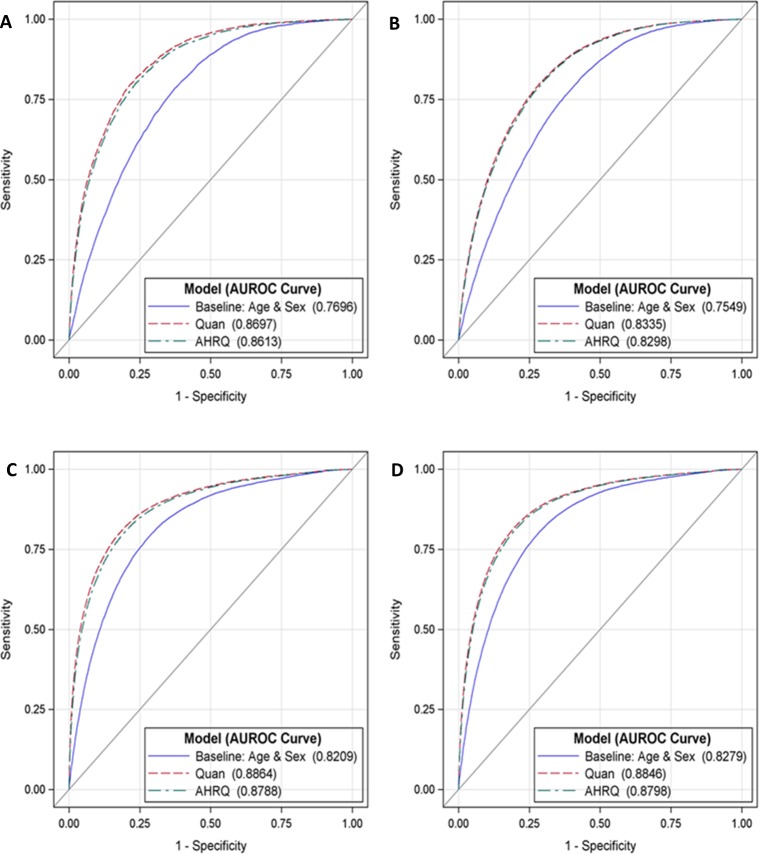
**AUROC comparisons by ECM for predicting inhospital mortality at index [A] and at 1 year [B] in high risk patients, and inhospital mortality at index [C] and at 1 year [D] in low risk patients.** High risk patients had one or more inpatient stay in the 12 months preceding the index encounter or three or more emergency department visits in the 3 months preceding the index encounter. Patients that did not satisfy the high-risk criteria were assigned to the low risk group. ROC = receiver operating characteristic, AUROC = area under the receiver operating characteristic.

Table 3 reports the performance measures of discrimination and calibration for the study sample and by patient risk groups. When predicting inhospital mortality during the index encounter, the Quan model (c = 0.887, 95% CI: 0.885–0.889) had negligible but significantly higher discrimination than the AHRQ (c = 0.880, 95% CI: 0.878–0.882) and baseline (c = 0.820, 95% CI: 0.818–0.822) models, p < .0001. Similar results were obtained for predicting inhospital mortality at 1-year, the discrimination performance of the Quan model (c = 0.884, 95% CI: 0.883–0.886) marginally exceeded the discrimination performance of the AHRQ (c = 0.880, 95% CI: 0.878–0.881) and baseline (c = 0.826, 95% CI: 0.824–0.827) models, p < .0001. Model discrimination for the mortality outcomes remained strong (AUROC>0.8) and the observed advantage of the Quan ECM over the AHRQ ECM was confirmed in both risk group samples. ROC plots displaying the minor discrimination advantage of the Quan ECM over the AHRQ ECM are provided in Figs 1 and 2.

**Table 3:** Measures of discrimination and calibration performance by ECM and mortality outcome.

		External Validation			
		Inhospital Mortality at Index		Inhospital Mortality at 1 Year	
		Quan	AHRQ	Quan	AHRQ
**All Patients N = 3,273,298**	AUROC ^a^ (95% CI)	0.887 (0.885,0.889) ^G^	0.880 (0.878,0.882) ^G^	0.884 (0.883,0.886) ^G^	0.880 (0.878,0.881) ^G^
	HL Test ^b^	485.5*	459.8*	890.1*	879.1*
	Brier Score ^c^	0.009	0.009	0.014	0.014
	R^2^ ^d^	24.9	23.1	25.1	24.0
	NRI>0 ^e^ (95% CI)	0.6115 (0.6006,0.6224)*		0.5234 (0.5149,0.5318)*	
	Reclassification, E—NE ^F^	-18%–79%		-27%–80%	
	Deaths (%)	31,298 (1.0)		50,215 (1.5)	
**High Risk Patients ^h^ N = 262,382**	AUROC ^a^ (95% CI)	0.870 (0.865,0.874) ^G^	0.861 (0.857,0.866) ^G^	0.834 (0.830,0.837) ^G^	0.830 (0.826,0.833) ^G^
	HL Test ^b^	106.6*	96.7*	271.5*	261.1*
	Brier Score ^c^	0.018	0.018	0.037	0.037
	R^2^ ^d^	22.7	21.4	20.7	20.0
	NRI>0 ^e^ (95% CI)	0.4725 (0.4450,0.5000)*		0.3566 (0.3384,0.3747)*	
	Reclassification, E—NE ^F^	-15%–62%		-28%–64%	
	Deaths (%)	5,035 (1.9)		11,043 (4.2)	
**Low Risk Patients ^h^ N = 3,010,916**	AUROC ^a^ (95% CI)	0.886 (0.884,0.889) ^G^	0.879 (0.877,0.881) ^G^	0.885 (0.883,0.886) ^G^	0.880 (0.878,0.882) ^G^
	HL Test ^b^	378.0*	410.6*	619.4*	616.2*
	Brier Score ^c^	0.008	0.008	0.012	0.012
	R^2^ ^d^	24.8	22.9	25.0	23.8
	NRI>0 ^e^ (95% CI)	0.6183 (0.6064,0.6302)*		0.5384 (0.5288,0.548)*	
	Reclassification, E—NE ^F^	-18%–80%		-27%–80%	
	Deaths (%)	26,263 (0.9)		39,172 (1.3)	

* P-value < 0.001. E = Events. NE = Non-events.

^a^ Area under the Receiver Operating Characteristic (ROC) curve (AUROC). AUROC is a measure of discrimination ranging from 0.5 (zero discrimination) to 1.0 (perfect discrimination).

^b^ Pearson chi-square value derived from the Hosmer–Lemeshow goodness-of-fit test [32].

^c^ Measure of predictive accuracy, greater accuracy is reflected by lower score.

^d^ R-squared, explained variation, displayed in percentage.

^e^ Category-free net reclassification improvement using the AHRQ ECM as the reference model.

^f^ E–NE, percentage of events (E) and non-events (NE) correctly reclassified by the Quan ECM compared to the AHRQ ECM.

^g^ AUROC curve differed significantly from the baseline model limited to age and sex (*p < 0*.*0001)*, and from the competing ECM (*p < 0*.*0001)*. Differences between AUROC curves were evaluated with the Mann-Whitney U test approach developed by DeLong et al. [35]. In the unstratified sample, the baseline model had an AUROC of 0.820 (95% CI 0.818–0.822) for inhospital mortality at index, and 0.826 (95% CI 0.824–0.827) for inhospital mortality at 1 year. For high risk patients, the baseline model had an AUROC of 0.770 (95% CI 0.764–0.775) for inhospital mortality at index, and 0.755 (95% CI 0.751–0.759) for inhospital mortality at 1 year. For low risk patients, the baseline model had an AUROC of 0.821 (95% CI 0.819–0.823) for inhospital mortality at index, and 0.828 (95% CI 0.826–0.830) for inhospital mortality at 1 year.

^h^ High risk patients had one or more inpatient stay in the 12 months preceding the index encounter or three or more emergency department visits in the 3 months preceding the index encounter. Patients that did not satisfy the high-risk criteria were assigned to the low risk group.

**Fig 3:**
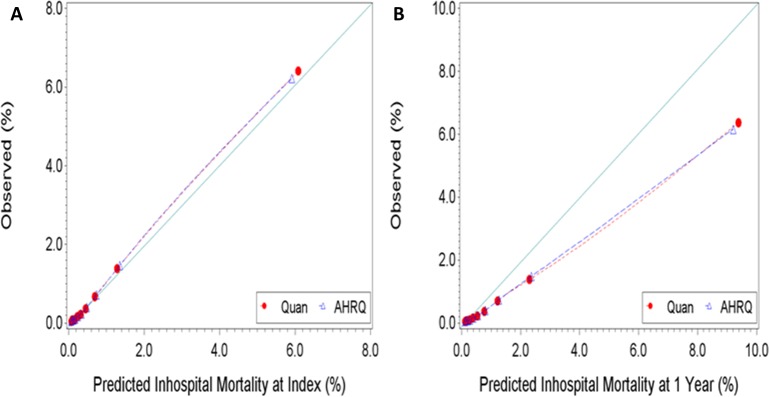
**Observed versus predicted risk of inhospital mortality [A] at index and [B] at 1 Year.** Perfect calibration is represented by the full line with a slope of 1 starting at the origin.

**Fig 4:**
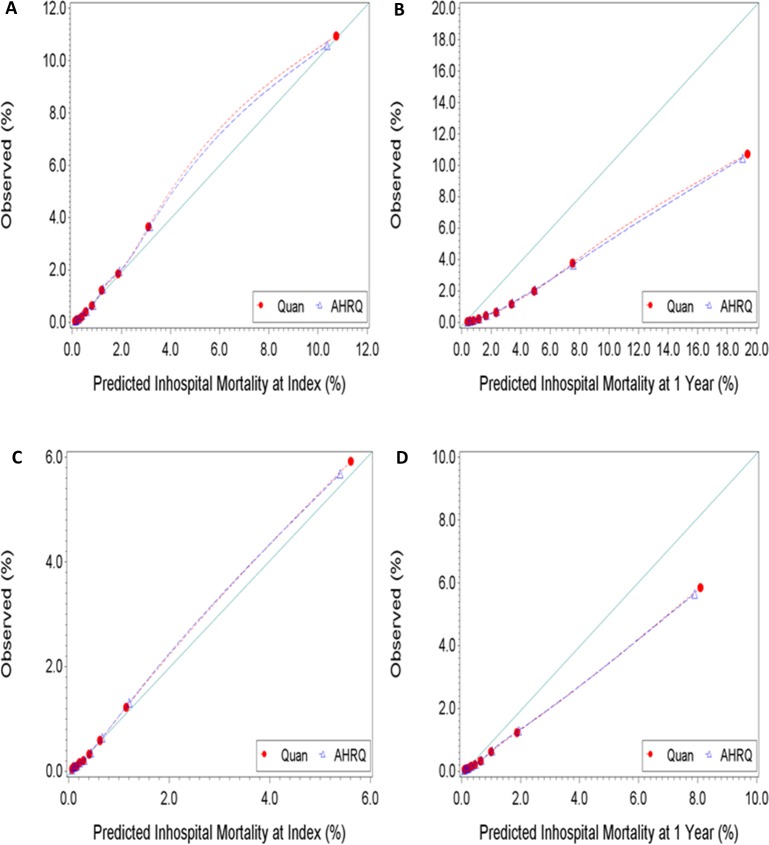
**Observed versus predicted risk of inhospital mortality at index [A] and at 1 year [B] for high risk patients, and inhospital mortality at index [C] and at 1 year [D] for low risk patients.** Perfect calibration is represented by the full line with a slope of 1 starting at the origin. High risk patients had one or more inpatient stay in the 12 months preceding the index encounter or three or more emergency department visits in the 3 months preceding the index encounter. Patients that did not satisfy the high-risk criteria were assigned to the low risk group.

There were no differences in Brier scores between competing ECMs, irrespective of the predicted outcome. The Brier scores were consistently lower when predicting inhospital mortality at index than at 1 year. These findings might indicate that the ECMs have better calibration when predicting the former outcome than when predicting the latter. The calibration plots reported in Figs 3 and 4 show good agreement between predicted and observed risk of inhospital mortality at index. However, the level agreement between the predicted and observed risk of inhospital mortality at 1 year were less satisfactory, suggesting the need for recalibration. As the observed risk of mortality at 1 year increased, the ECMs increasingly over-predicted the outcome. Results from the Hosmer–Lemeshow goodness-of-fit test indicated imperfect agreement between expected and observed risk, irrespective of the ECM-outcome combination assessed. This is expected given the large study sample and previously reported simulation results from Kramer et al. [36] showing the Hosmer-Lemeshow test to be particularly sensitive to sample size: even with a minor deviation (0.4%) from perfect fit between expected and observed risk, studies with sample sizes of 50,000 or more observations rejected the null hypothesis 100% of the time.

Explained variation (R^2^) was consistently higher, by approximately 1 to 2% with the Quan ECM compared to the AHRQ ECM across patient groups and mortality outcomes. Measures of discrimination (AUROC), calibration (Brier scores), and overall performance (R^2^) were consistently better in the low risk patient group compared to the high risk patient group.

Net reclassification improvements were observed by the Quan ECM over the AHRQ ECM for the full sample, in high and low risk patients, and in patients with index encounter limited to inpatient stays. However, the magnitude of these improvements was low to moderate, between 0.35 to 0.62, on a possible NRI range of -2 to 2. The positive NRI scores observed, and the apparent greater predictive performance of the Quan ECM, were driven principally by improvements in model specificity (the correct reclassification of non-events). In every sample-outcome combination examined, the percentage of events correctly reclassified by the Quan ECM was negative, possibly indicating reduced sensitivity compared to the AHRQ ECM. In the sample limited to persons whose index encounter was an emergency department visit, this reduced sensitivity combined with minimal improvement in specificity by the Quan ECM compared to the AHRQ ECM resulted in negative NRI.

### Sensitivity analyses

Results from the sensitivity analyses are available as supplementary material (S1 Table, S1 and S2 Figs). As hypothesized, patients whose index encounter was an inpatient stay had a greater risk of inhospital mortality at index [2.4% vs 0.3%, χ2 (1, N = 3,273,298) = 33,138.7, p < .0001], and at 1 year [3.6% vs 0.6%, χ2 (1, N = 3,273,298) = 43,585.6, p < .0001] than patients whose index encounter was an ED visit. Performance measures in the analyses stratified by index encounter type mimicked the trends reported for the unstratified sample, except for explained variation which was 48 to 69 percent lower in the ED visit sample than in the inpatient sample. Measures of discrimination (AUROC) and calibration (Brier scores) were superior in the inpatient sample compared to the ED visit sample.

## Discussion

We conducted an external validation and compared the ability of the Quan and AHRQ ECMs to predict inhospital mortality at index and at 1-year in the Cerner HF database. In a prior study, the Quan ECM demonstrated superior predictive performance over the AHRQ version 3.0 ECM for inhospital mortality at index in a Canadian population with universal health coverage [18]. The current study expands on prior findings and demonstrates the performance advantage of the Quan ECM over the AHRQ version 3.7 ECM for inhospital mortality at index and at 1-year in Cerner Health Facts®. This is the first study to evaluate any diagnostic-based risk adjustment methods in HF and to confirm the excellent discrimination performance of both the Quan and AHRQ ECMs in a multi-payer US health data source. While significant, increased discrimination performance of the Quan ECM over the AHRQ ECM did not exceed 1% for any of the mortality outcomes after the inclusion of baseline variables age and sex. It is therefore unlikely that the observed differences between the ECMs are clinically meaningful. The marginal performance improvement in discrimination and explained variance observed between the ECMs may be a consequence of the large sample available for analysis and might not be reproducible in smaller HF subsets or patient subpopulations. In this study, evidence of superior predictive performance by the Quan ECM was demonstrated in an undifferentiated patient population, in patient groups stratified by risk of hospital readmission and death, and in patients stratified by their index encounter type [ED visits and inpatient stays].

Visual inspection of the calibration plots for the Quan and AHRQ ECMs revealed noticeable levels of disagreement between predicted and observed risk of inhospital mortality at 1 year. Lower calibration performance appeared more pronounced in the high risk patient group compared to the low risk patient group. To improve accuracy, we recommend that the ECMs be recalibrated specifically for predictions of inhospital mortality at 1 year in HF. The observed over-prediction of inhospital mortality at 1 year by the ECMs likely results from a combination of factors ranging from suboptimal parameter section to outcome misclassification.

The ICD-9 codes used to assess the prevalence of comorbid conditions by the competing ECMs resulted in minimal variations in disease frequencies; prevalence differed by less than 1% for the majority of conditions. The exclusion of cardiac arrhythmia from the AHRQ ECM may be responsible for the observed predictive performance differences. In the Quan ECM, cardiac arrhythmia was the fifth most prevalent condition and was significantly associated with increased odds of inhospital mortality at index and at 1-year in the adjusted models (S2 Table).

This study has limitations. Like other EHR-derived data warehouse used for observational research that comply with the U.S. HIPAA law and regulations, HF adheres to de-identification procedures that prevent further linkage to registries such as the National Death Index and other health organizations outside the same HIPAA-covered entity. Since deaths were limited to those recorded during inpatient care and within HIPAA-covered networks, some misclassification of mortality at 1-year is to be expected from the deaths that occurred outside these settings. The patient de-identification process also implies that the diagnoses and combination of ICD codes used for estimating ECM prevalence could not be validated using chart re-abstraction methodology leaving doubts about their sensitivity and specificity in HF. Limiting the assessment of morbidities to a single index encounter, as opposed to including a look back period in the assessment of a patient’s health, likely resulted in the misclassification of previously diagnosed health conditions as absent. Including a look back periods of one to two years generally improves the detection of prevalent health conditions [45, 46]. One explanation for this improvement is that look back periods limit bias in discharge abstract coding whereby secondary health conditions tend to be under recorded in patients treated for severe acute conditions and vice versa [15, 47]. A look back period was not included in this study to increase the comparability of results with the original Quan [18] paper and because our research group is currently conducting a parallel study to test the consequences of varying look back periods in HF.

HF was not primarily designed for research purposes [1]. Study findings are therefore subject to the same risks, biases, and limitations typically associated with research based on electronic health data [48, 49]. These include potential for selection bias, missing or incomplete documentation, coding errors, misclassifications of diagnostic codes, record linkage errors due to interoperability issues, and duplication. Finally, recorded comorbid conditions could not be separated from conditions resulting from complications in care. Thus, it was impossible to evaluate the effects of excluding complications of care from our models.

The Quan and the AHRQ (version 3.7) ECMs were found to be practically equivalent in discriminating between short- and long-term inhospital mortality outcomes in HF. While ECM calibration measures were satisfactory for predicting inhospital mortality at index, recalibration of the ECMs is recommended to improve the predictive accuracy for inhospital mortality at 1 year. These diagnostic-based risk adjustment tools should enhance capacity for conducting quality observational studies and health services research using Health Facts® data.

## Supporting information

S1 Fig **AUROC comparison by ECM for predicting inhospital mortality at index [A] and at 1 Year [B] for index encounters limited to emergency department visits, and inhospital mortality at index [C] and at 1 Year [D] for index encounters limited to inpatient stays.** AUROC = area under the receiver operating characteristic, ROC = receiver operating characteristic.
(TIF)

S2 Fig **Observed versus predicted risk of inhospital mortality at index [A] and at 1 Year [B] for index encounters limited to emergency department visits, and inhospital mortality at index [C] and at 1 year [D] for index encounters limited to inpatient stays.** Perfect calibration is represented by the full line with a slope of 1 starting at the origin.
(TIF)

S1 Table Measures of discrimination and calibration performance for inhospital mortality by index encounter type, ED visits and inpatient stays. * P-value < 0.001. ED = Index encounter is an emergency department visit. IS = Index encounter is an inpatient stay. E = Events. NE = Non-events. ^a^ Area under the Receiver Operating Characteristic (ROC) curve (AUROC). AUROC is a measure of discrimination ranging from 0.5 (zero discrimination) to 1.0 (perfect discrimination). ^b^ Pearson chi-square value derived from the Hosmer–Lemeshow goodness-of-fit test [32]. ^c^ Measure of predictive accuracy, greater accuracy is reflected by lower score. ^d^ Generalized R-squared, explained variation, displayed in percentage. ^e^ Category-free net reclassification improvement with the AHRQ ECM as the reference model. ^f^ E–NE, percentage of events (E) and non-events (NE) correctly reclassified by the Quan ECM compared to the AHRQ ECM. ^g^ AUROC curve differed significantly from the baseline model limited to age and sex (*p < 0*.*0001)*, and from the competing ECM (*p < 0*.*0001)*. Differences between AUROC curves were evaluated with the Mann-Whitney U test approach developed by DeLong et al. (1988). For ED encounters, the baseline model had an AUROC of 0.804 (95% CI 0.799–0.810) for inhospital mortality at index, and 0.826 (95% CI 0.822–0.829) for inhospital mortality at 1 year. For IS encounters, the baseline model had an AUROC of 0.752 (95% CI 0.749–0.754) for inhospital mortality at index, and 0.754 (95% CI 0.752–0.756) for inhospital mortality at 1 year.
(DOCX)

S2 Table Adjusted odds ratios (95% CI) of Elixhauser conditions for inpatient mortality at index and at 1-year by ECM variant, N = 3,273,298. Odds ratios are adjusted for baseline variables sex and age. The odds ratios reported are those that reached statistical significance (p<0.05). Abbreviation: CI, confidence intervals.
(DOCX)
