# Supplementary figures and images for: External validation and comparison of two variants of the Elixhauser comorbidity measures for all-cause mortality
Source: PLoS One. 2017 Mar 28;12(3):e0174379. doi: 10.1371/journal.pone.0174379 (PMC5369776; doi:10.1371/journal.pone.0174379)

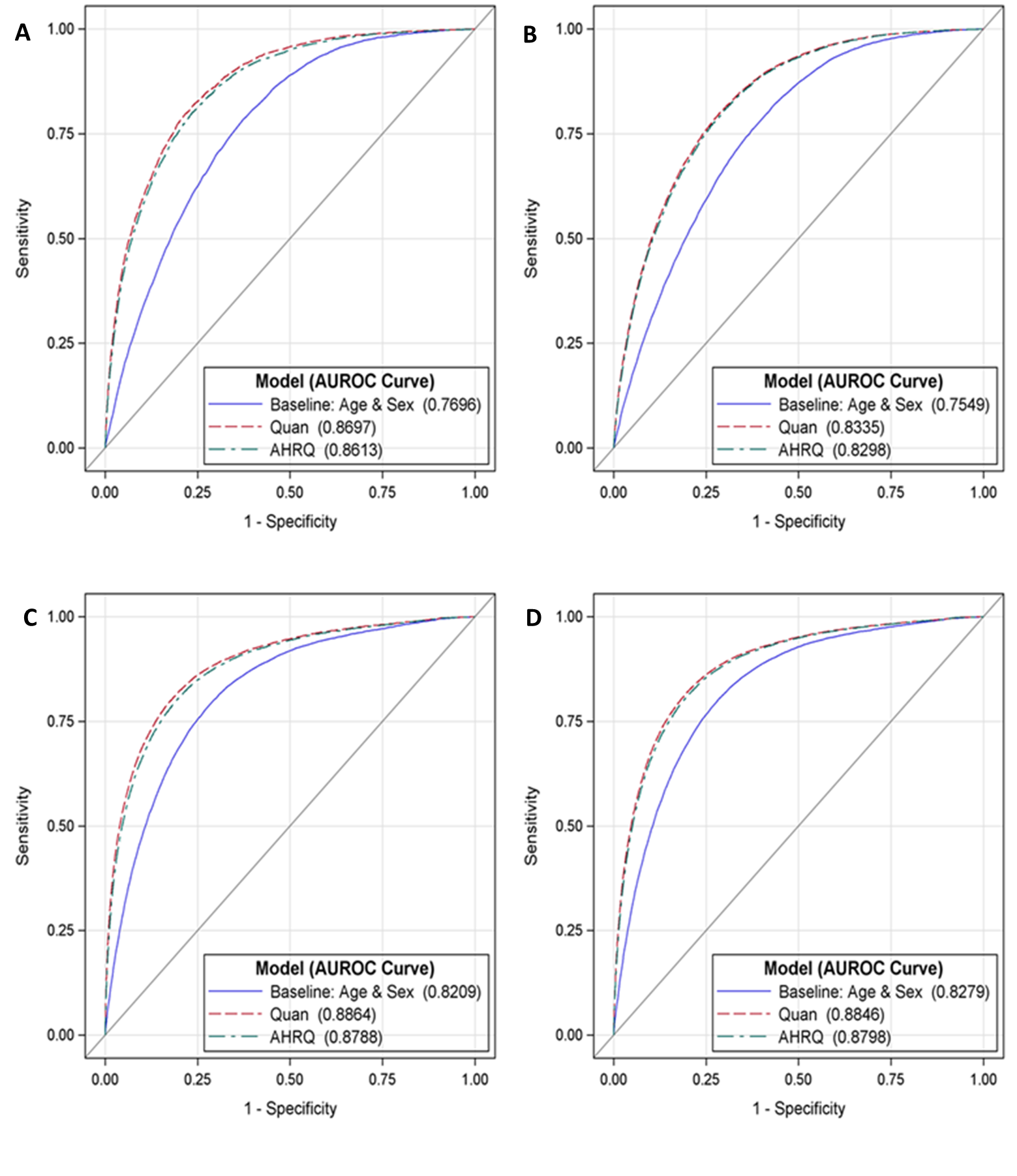

Supplement: S1 Fig — AUROC comparison by ECM for predicting inhospital mortality at index [A] and at 1 Year [B] for index encounters limited to emergency department visits, and inhospital mortality at index [C] and at 1 Year [D] for index encounters limited to inpatient stays. AUROC = area under the receiver operating characteristic, ROC = receiver operating characteristic. (TIF) [file pone.0174379.s001.tif]

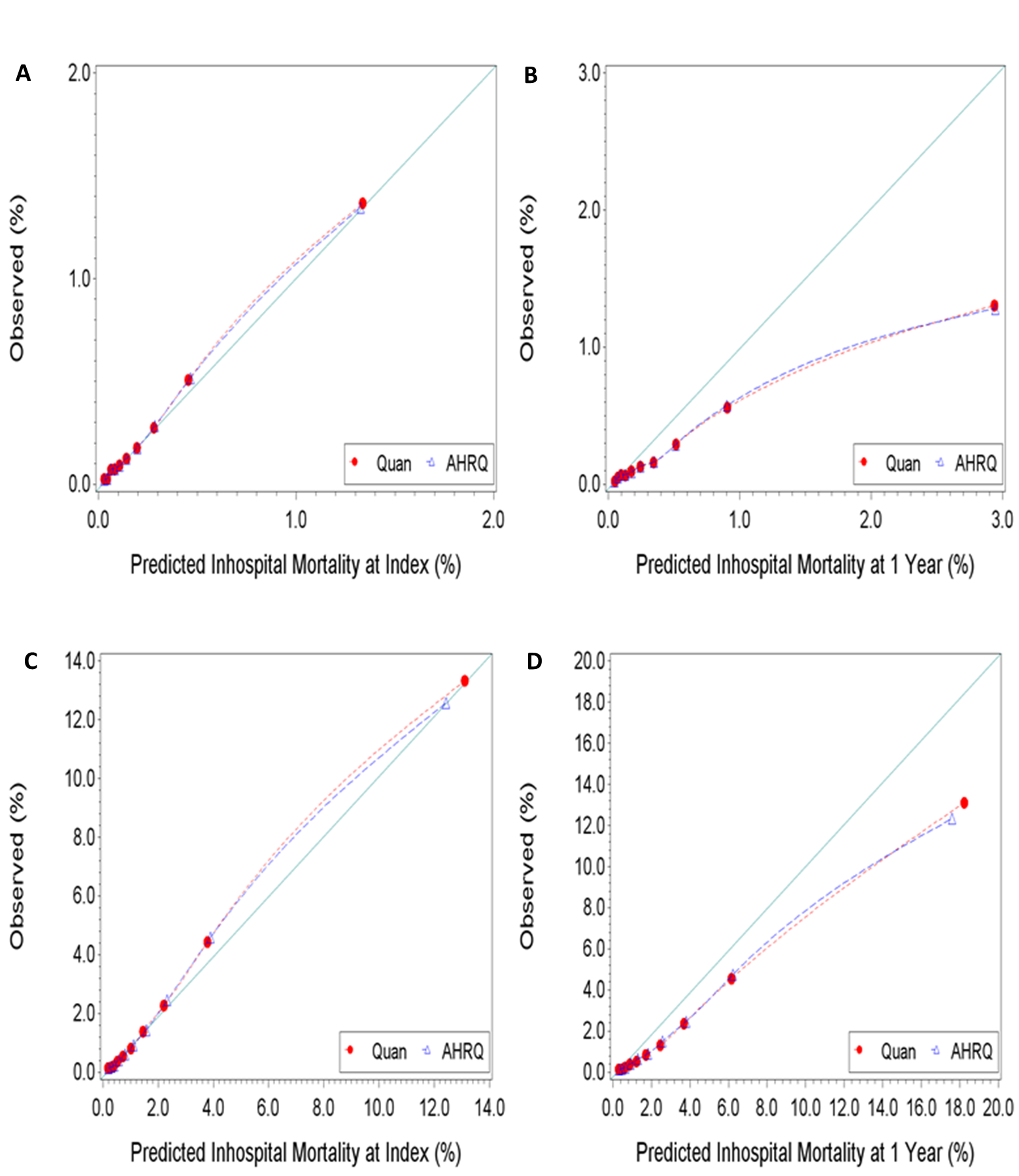

Supplement: S2 Fig — Observed versus predicted risk of inhospital mortality at index [A] and at 1 Year [B] for index encounters limited to emergency department visits, and inhospital mortality at index [C] and at 1 year [D] for index encounters limited to inpatient stays. Perfect calibration is represented by the full line with a slope of 1 starting at the origin. (TIF) [file pone.0174379.s002.tif]
