# Supplementary material for: External validation and comparison of two variants of the Elixhauser comorbidity measures for all-cause mortality
Source: PLoS One. 2017 Mar 28;12(3):e0174379. doi: 10.1371/journal.pone.0174379 (PMC5369776; doi:10.1371/journal.pone.0174379)
Supplement: S1 Table — * P-value < 0.001. ED = Index encounter is an emergency department visit. IS = Index encounter is an inpatient stay. E = Events. NE = Non-events. a Area under the Receiver Operating Characteristic (ROC) curve (AUROC). AUROC is a measure of discrimination ranging from 0.5 (zero discrimination) to 1.0 (perfect discrimination). b Pearson chi-square value derived from the Hosmer–Lemeshow goodness-of-fit test [32]. c Measure of predictive accuracy, greater accuracy is reflected by lower score. d Generalized R-squared, explained variation, displayed in percentage. e Category-free net reclassification improvement with the AHRQ ECM as the reference model. f E–NE, percentage of events (E) and non-events (NE) correctly reclassified by the Quan ECM compared to the AHRQ ECM. g AUROC curve differed significantly from the baseline model limited to age and sex (p < 0.0001), and from the competing ECM (p < 0.0001). Differences between AUROC curves were evaluated with the Mann-Whitney U test approach developed by DeLong et al. (1988). For ED encounters, the baseline model had an AUROC of 0.804 (95% CI 0.799–0.810) for inhospital mortality at index, and 0.826 (95% CI 0.822–0.829) for inhospital mortality at 1 year. For IS encounters, the baseline model had an AUROC of 0.752 (95% CI 0.749–0.754) for inhospital mortality at index, and 0.754 (95% CI 0.752–0.756) for inhospital mortality at 1 year. (DOCX) [file pone.0174379.s003.docx]

**S1 Table: Measures of discrimination and calibration performance for inhospital mortality by index encounter type, ED visits and inpatient stays.**

|  |  | External Validation | | | | |  |
| --- | --- | --- | --- | --- | --- | --- | --- |
|  |  | **Inhospital Mortality at Index** | | **Inhospital Mortality at 1 Year** | | |  |
|  |  | **Quan** | **AHRQ** | **Quan** | **AHRQ** |  |  |
| ED N=2,204,680 (67.4%) | AUROC ^a^ (95% CI) | 0.816 (0.811,0.822) ^g^ | 0.813 (0.807,0.818) ^g^ | 0.839 (0.835,0.842) ^g^ | 0.838 (0.834,0.842) ^g^ | | |
|  | HL Test ^b^ | 48.6* | 46.8* | 79.5* | 76.0* | | |
|  | Brier Score ^c^ | 0.003 | 0.003 | 0.005 | 0.005 | | |
|  | R^2 d^ | 11.0 | 10.7 | 14.8 | 14.7 | | |
|  | NRI>0 ^e^ (95% CI) | -0.5131 (-0.5335,-0.4927)* | | -0.3639 (-0.3778,-0.3499)* | | | |
|  | Reclassification, E - NE ^F^ | -59% - 8% | | -63% - 26% | | | |
|  | Deaths (%) | 6,051 (0.27) | | 12,053 (0.55) | | | |
| IS 1,068,618 (32.6%) | AUROC ^a^ (95% CI) | 0.862 (0.860,0.864) ^g^ | 0.849 (0.847,0.852) ^g^ | 0.851 (0.849,0.853) ^g^ | 0.843 (0.841,0.845) ^g^ | | |
|  | HL Test ^b^ | 417.0* | 397.3* | 924.5* | 895.7* | | |
|  | Brier Score ^c^ | 0.021 | 0.021 | 0.031 | 0.032 | | |
|  | R^2 d^ | 23.1 | 20.9 | 22.8 | 21.4 | | |
|  | NRI>0 ^e^ (95% CI) | 0.5033 (0.4908,0.5157)* | | 0.4242 (0.4142,0.4343)* | | | |
|  | Reclassification, E - NE ^F^ | -5% - 55% | | -13% - 55% | | | |
|  | Deaths (%) | 25,247 (2.36) | | 38,162 (3.57) | | | |

* P-value < 0.001.

^a^ Area under the Receiver Operating Characteristic (ROC) curve (AUROC). AUROC is a measure of discrimination ranging from 0.5 (zero discrimination) to 1.0 (perfect discrimination).

^b^ Pearson chi-square value derived from the Hosmer–Lemeshow goodness-of-fit test [[32](#_ENREF_32)].

^c^ Measure of predictive accuracy, greater accuracy is reflected by lower score.

^d^ Generalized R-squared, explained variation, displayed in percentage.

^e^ Category-free net reclassification improvement with the AHRQ ECM as the reference model.

^f^ E – NE, percentage of events (E) and non-events (NE) correctly reclassified by the Quan ECM compared to the AHRQ ECM.

^g^ AUROC curve differed significantly from the baseline model limited to age and sex (*p < 0.0001),* and from the competing ECM (*p < 0.0001)*. Differences between AUROC curves were evaluated with the Mann-Whitney U test approach developed by DeLong et al. (1988). For ED encounters, the baseline model had an AUROC of 0.804 (95% CI 0.799-0.810) for inhospital mortality at index, and 0.826 (95% CI 0.822-0.829) for inhospital mortality at 1 year. For IS encounters, the baseline model had an AUROC of 0.752 (95% CI 0.749-0.754) for inhospital mortality at index, and 0.754 (95% CI 0.752-0.756) for inhospital mortality at 1 year.

**Abbreviations:** E, events; NE, non-events; ED, index encounter is an emergency department visit; IS, index encounter is an inpatient stay.
