# Supplementary material for: External validation and comparison of two variants of the Elixhauser comorbidity measures for all-cause mortality
Source: PLoS One. 2017 Mar 28;12(3):e0174379. doi: 10.1371/journal.pone.0174379 (PMC5369776; doi:10.1371/journal.pone.0174379)
Supplement: S2 Table — Odds ratios are adjusted for baseline variables sex and age. The odds ratios reported are those that reached statistical significance (p<0.05). Abbreviation: CI, confidence intervals. (DOCX) [file pone.0174379.s004.docx]

**S2 Table. Adjusted odds ratios (95% CI) of Elixhauser conditions for inpatient mortality at index and at 1-year by ECM variant, N= 3,273,298.**

|  | Inpatient Mortality at Index at Index | | Inpatient Mortality at 1-Year | |
| --- | --- | --- | --- | --- |
| Condition | **Quan** | **AHRQ** | **Quan** | **AHRQ** |
| Congestive Heart Failure | 2.29 (2.22-2.37) | 2.66 (2.57-2.75) | 2.29 (2.23-2.35) | 2.55 (2.49-2.62) |
| Cardiac Arrhythmia | 1.81 (1.76-1.87) | Not applicable | 1.51 (1.47-1.54) | Not applicable |
| Valvular Disease | 0.73 (0.69-0.77) | 0.88 (0.82-0.95) | 0.84 (0.81-0.88) | Not-significant |
| Pulmonary Circulation Disorders | 1.51 (1.42-1.62) | 1.58 (1.48-1.68) | 1.37 (1.29-1.44) | 1.54 (1.47-1.63) |
| Peripheral Vascular Disorders | 1.42 (1.35-1.50) | 1.54 (1.47-1.62) | 1.41 (1.35-1.47) | 1.49 (1.43-1.55) |
| Hypertension Combined | 0.54 (0.52-0.55) | 0.55 (0.54-0.57) | 0.64 (0.62-0.65) | 0.65 (0.63-0.66) |
| Paralysis | 3.40 (3.16-3.66) | 3.39 (3.18-3.61) | 2.97 (2.78-3.17) | 2.86 (2.71-3.02) |
| Other Neurological Disorders | 3.77 (3.63-3.91) | 2.12 (2.04-2.20) | 2.90 (2.81-3.00) | 1.85 (1.80-1.92) |
| Chronic Pulmonary Disease | 1.09 (1.05-1.12) | 1.17 (1.13-1.21) | 1.35 (1.31-1.38) | 1.41 (1.38-1.45) |
| Diabetes Uncomplicated | 0.87 (0.84-0.90) | 0.88 (0.85-0.91) | Not-significant | Not-significant |
| Diabetes Complicated | 0.71 (0.65-0.77) | 0.71 (0.66-0.77) | 1.09 (1.03-1.15) | 1.09 (1.03-1.15) |
| Hypothyroidism | 0.63 (0.60-0.67) | 0.67 (0.64-0.71) | 0.72 (0.69-0.75) | 0.74 (0.71-0.77) |
| Renal Failure | 1.77 (1.70-1.85) | 1.85 (1.77-1.93) | 2.03 (1.97-2.10) | 2.03 (1.96-2.10) |
| Liver Disease | 3.69 (3.50-3.90) | 2.72 (2.54-2.90) | 3.57 (3.41-3.74) | 3.12 (2.95-3.29) |
| Peptic Ulcer Dis. excl. bleeding | 0.39 (0.32-0.49) | Not-significant | 0.63 (0.54-0.73) | Not-significant |
| AIDS/HIV | 3.22 (2.72-3.82) | 3.77 (3.19-4.45) | 3.91 (3.41-4.48) | 4.34 (3.80-4.97) |
| Lymphoma | 2.04 (1.83-2.27) | 2.13 (1.92-2.38) | 2.86 (2.63-3.11) | 2.90 (2.67-3.15) |
| Metastatic Cancer | 3.22 (3.03-3.42) | 3.35 (3.16-3.56) | 3.50 (3.33-3.67) | 3.60 (3.43-3.77) |
| Solid Tumor without Metastasis | 1.58 (1.50-1.66) | 1.60 (1.52-1.68) | 1.95 (1.87-2.03) | 1.95 (1.87-2.03) |
| Rheumatoid Arthritis/collagen | Not-significant | Not-significant | 1.20 (1.12-1.29) | 1.17 (1.08-1.26) |
| Coagulopathy | 2.74 (2.61-2.87) | 3.35 (3.19-3.51) | 2.39 (2.29-2.49) | 2.73 (2.62-2.85) |
| Obesity | 0.88 (0.82-0.94) | Not-significant | 0.88 (0.83-0.93) | 0.91 (0.86-0.96) |
| Weight Loss | 2.53 (2.41-2.66) | 2.63 (2.5-2.78) | 2.35 (2.25-2.46) | 2.40 (2.29-2.51) |
| Fluid and Electrolyte Disorders | 3.20 (3.11-3.29) | 3.60 (3.51-3.71) | 2.44 (2.39-2.50) | 2.63 (2.57-2.70) |
| Blood Loss Anemia | Not-significant | 0.84 (0.75-0.95) | Not-significant | Not-significant |
| Deficiency Anemia | 0.43 (0.38-0.48) | 0.66 (0.63-0.69) | 0.67 (0.62-0.73) | 0.89 (0.86-0.92) |
| Alcohol Abuse | Not-significant | 1.13 (1.06-1.20) | 1.15 (1.10-1.21) | 1.19 (1.13-1.26) |
| Drug Abuse | Not-significant | 1.27 (1.15-1.40) | Not-significant | 1.23 (1.13-1.33) |
| Psychoses | 0.73 (0.65-0.82) | 0.64 (0.58-0.70) | Not-significant | 0.79 (0.74-0.85) |
| Depression | 0.68 (0.64-0.73) | 0.75 (0.70-0.81) | 0.80 (0.76-0.84) | 0.86 (0.82-0.91) |

**Note:** Odds ratios are adjusted for baseline variables sex and age. The odds ratios reported are those that reached statistical significance (p<0.05).

**Abbreviation:** CI, confidence intervals.
